# A Blockchain-Based Authorization System for Trustworthy Resource Monitoring and Trading in Smart Communities

**DOI:** 10.3390/s18103561

**Published:** 2018-10-20

**Authors:** Ramon Alcarria, Borja Bordel, Tomás Robles, Diego Martín, Miguel-Ángel Manso-Callejo

**Affiliations:** 1 Departamento de Ingeniería Topográfica y Cartografía, Universidad Politécnica de Madrid, UPM Campus Sur, Km 7.5 de la Autovía de Valencia, 28031 Madrid, Spain; m.manso@upm.es; 2 Departamento de Ingeniería de Sistemas Telemáticos, Universidad Politécnica de Madrid, Avenida Complutense no. 30, 28040 Madrid, Spain; bbordel@dit.upm.es (B.B.); tomas.robles@upm.es (T.R.); diego.martin.de.andres@upm.es (D.M.)

**Keywords:** resource monitoring, prosumer, blockchain, trading, resource auction, smart communities, power consumption, water consumption

## Abstract

Resource consumption in residential areas requires novel contributions in the field of consumer information management and collaborative mechanisms for the exchange of resources, in order to optimize the overall consumption of the community. We propose an authorization system to facilitate access to consumer information and resource trading, based on blockchain technology. Our proposal is oriented to the Smart communities, an evolution of Community Energy Management Systems, in which communities are involved in the monitoring and coordination of resource consumption. The proposed environment allows a more reliable management of monitoring and authorization functions, with secure data access and storage and delegation of controller functions among householders. We provide the definition of virtual assets for energy and water resource sharing as an auction, which encourages the optimization of global consumption and saves resources. The proposed solution is implemented and validated in application scenarios that demonstrate the suitability of the defined consensus mechanism, trustworthiness in the level of provided security for resource monitoring and delegation and reduction on resource consumption by the resource trading contribution.

## 1. Introduction

Resource consumption in residential areas is a fundamental aspect in the field of Smart Cities, since it represents a significant part of the total consumption of resources in a city. New concepts such as Home Energy Management and Smart Metering have appeared, bringing together works that contribute to an optimization in the demand for resources from household inhabitants, also considering the variations in time of the price of these resources.

In residential buildings, there exists the concept of condominium and community management, in which the residential community is responsible for maintaining the environment, security and resource consumption. Communities negotiate contracts with resource supply companies such as energy and water for better conditions than the ones applied to individual householders. Thus, there is an evolution from Home Energy Management Systems (HEMS) to the new concept of Community Energy Management Systems (CEMS), which is defined as a smart grid-based approach based on a more efficient resource management, for the creation of self-sustaining or more sustainable communities, to lessen the impact on the environment.

To make an optimal resource distribution among the inhabitants, it is necessary to know the particular needs of each home in real time and adapt or distribute the consumption to cover those demands. These systems are based on the need to manage information from a smart home, through its networks of intelligent devices, consisting of sensors and actuators. With this information, consumers communities can flexibly absorb periods of peak loads from individual consumers. In this way, the energy needs of the community are stabilized, allowing a better planning of contracted resources. To cover these demands, the concept of resource trading arises [1], by which users who have contracted a certain resource quota lend or borrow resources from their neighbours, in turn using “resource credits” as currency.

In addition, access to information on the performance of a home at resource consumption level must be protected, both by access from outside and by unauthorized access from other homes belonging to the smart community. Some problems have appeared in recent years with smart lights [2], although a more critical problem could be found in the unauthorized access to information on devices that manage the security of the house such as smart doors or windows, or systems of surveillance such as cameras. The smart community must also have the proper mechanisms for authorization and management of access privileges over common and private resources, to allow the delegation of monitoring functions among neighbours, for example with the care of animals, surveillance tasks or monitoring of the correct operation of devices that have been connected during the absence of neighbours.

The blockchain technology [3], as an information exchange platform used to secure transactions with cryptocurrencies, can in turn be used to provide a flexible and secure platform to allow the provision of sensitive transactions, with privacy and reliability components enabling trust [4].

The objective of this paper is the provision of a blockchain-based acquisition system for the monitoring of information produced by the networks of smart devices installed in smart communities. We therefore propose the use of blockchain technology to provide security and reliability to the consumption of monitored resources and in turn, allow resource trading through the management of transactions in a defined resource credit system. As specific contributions, the first one is an authorization model for the access of the network participants to the consumption information coming from the devices belonging to the sensor networks. By this proposal a trustworthy authorization mechanism is provided to enable monitoring of resource consumption by the system’s manager, the securitization of data stored in the blockchain and the delegation of controller functions among householders.

The second main contribution is a trustworthy resource trading proposal, considering a token model exclusively designed for operations within the smart community and resource trading mechanism based on auctions adapted to the management of cryptocurrencies.

The structure of the paper is as follows: Section 2 presents the state of the art on resource monitoring in smart homes and blockchain technology for resource coordination and trading. Section 3 describes the functional components required for the formalization of the solutions and the supporting architecture for smart communities. The main contributions of our work, that is, the abstract model for the authorization system and the trustworthy resource sharing are described in Section 4 and Section 5 respectively. The system implementation and some experiments and results are provided in Section 6 and Section 7. Finally, Section 8 shows the conclusions and future work.

## 2. State of the Art

In this section, we present the state of the art related to monitoring of resources in smart communities, individual and collective resource consumption coordination and the use of blockchain for resource monitoring and trading. Finally, we also review the works that question security in blockchain networks and that contribute to solve the disadvantages of this technology.

In relation to the work on monitoring in smart homes, smart communities and ultimately in smart cities we can see how the monitoring and prediction of consumption in smart communities is a key issue that has not been resolved in the related works. Some works provide control techniques based on prioritization and configurable power limits [5], considering occupation and smart devices usage profiles. Other works focus on the calculation of power limits through probabilistic models [6] or disaggregation techniques [7]. In general, these works do not consider the complexity of the distribution of power limits among several users of a neighbourhood community.

For the study of resource consumption monitoring and prediction, where many rooms with several users are involved, we usually work with data acquisition techniques for modelling user’s behavioural predictions [8] and with the concept of CEMS, in which smart communities maintain sources of energy production such as solar panels and other individual and collective consumption sources, such as electric vehicle charging systems [9]. The current problems of collective consumption monitoring suffer from several problems. From the point of view of its flexibility and usability, it is generally operated by centralized systems that store all the information. The centralization of monitoring information presents security problems (by concentrating the probability of attacks and unauthorized access) and also a sense of privacy and ownership of information, since users prefer to have information about their devices in their own homes. In this paper, we present a resource monitoring and authorization mechanism for trustworthy resource provision and trading among participants in a smart community.

In order to maintain a stabilized demand for energy in a smart community, it is necessary to effectively coordinate individual consumption, thus allowing better planning of contracted resources. To cover this demand the concept of resource trading [10] arises, by which users who have contracted a certain quota of resources lend or borrow resources from their neighbours. To facilitate the exchange of this type of service, a monetary system of resource credits is created, based on the concept of resource assignment from collaborative cloud services [11]. This concept of resource trading also appears in other research works ranging from the exchange of energy [12,13] to the exchange of professional services, such as peer reviews [14], in the academic field. All these works define a brokerage trading system that values individual contributions and stores the merit accumulated by these users. The problem highlighted by these works is the degree of reliability offered by these systems to their users. Being centralized systems and generally controlled by third-parties, they are prone to attacks and integrity violations, which would affect the information contained and therefore, the trust between the involved devices [4].

Blockchain technology [3] allows that the information is stored across all computers and it can be opened by any peer possessing the “public key“. Modification however, is not allowed, even by the original author. Data records are timestamped in the form of transactions, providing a trusted and timed record of the added data. Thus, blockchain technology is optimal for resource monitoring and trading [15].

Some jobs are appearing in the consumer electronics sector for monitoring and accessing resources through well-stablished home automation companies such as Comcast Corporation [16]. Other start-ups such as Electron take advantage of the smart meters from millions of homes in the UK to allow users to cut their energy usage by using blockchain technology. It constitutes the first energy blockchain platform scaled to a national level, using data to simulate 53 million meters and 60 suppliers. Other works describe the use of blockchain for the monitoring of resources in the field of Insurance [17], Wireless Sensor Networks [18], computational task cooperation [19] and E-agriculture [20].

From the point of view of resource trading, blockchain technology allows the creation of tokens [21], that represent digital assets that can be used as currency for the provision of computational resources or other types of resources. There are several works using blockchain as a mean to incentive action, for example, the forwarding of packets in vehicular networks [22,23]. However, the analysed works do not contemplate the use of blockchain technology and specifically the use of tokens as a reward for the resource saving. In our work, we apply this objective to the field of smart communities.

In 2014 SolarCoin appears, a cryptocurrency earned by generating solar electricity. Inspired by Bitcoin and blockchain start-ups whereby transactions are recorded and validated by a peer to peer network of computers. The company provides an automated token system that pays people for the electricity they produce. In this project, the creation of cryptocurrency has an economic orientation (SolarCoin can be sent to a Bitcoin wallet and can also be converted into hard cash after). The same happens with other initiatives such as Plastic bank or RecycleToCoin, which set up collection centres where people can set down used plastic and aluminium cans for cryptocurrency. In our work, we use the concept of token, which being a reward oriented solely to the service of resource trading, allows us to operate under the same implementation of the deployed blockchain networks.

In relation to the creation of assets for the exchange of energy resources, Bergquist et al. [24] propose the definition of tokens for blockchain-based transactive microgrids. The concept of token usage by prosumers is incorporated but the dynamics of token estimation related to power consumption is not determined. Other works [25] provide blockchain tokenization as a mean to guarantee the origin of the energy, taking advantage of the certification capabilities of the network. Two price strategies are defined and simulated. Although the purpose of our work is not to certify the origin of the energy, we also take a condition-based approach by providing token conditions such as time interval or expiration date.

The revised initiatives additionally present a problem in the consensus procedure for the growth of the network. Most blockchain implementations (such as Bitcoin or Ethereum) consider the so-called Proof of Work (PoW) [26], where some network participants called miners compete against each other to complete transactions on the network and get rewarded. The competition consists of solving a complicated mathematical puzzle which requires massive computing capabilities. PoW is energy consumption intensive, thus, it is not very appealing when we discuss energy efficiency [10]. Therefore, we propose an alternative version based on Proof of Authority (PoA) [27], where instead of miners racing to find a solution to a difficult problem, authorized signers create new blocks based on some proposed rules.

Finally, there are other alternatives that offer the concept of microgrid, through which neighbours can locally generate and trade their energy, keeping record of energy consumption and generation by each household. These approaches are Lo3 Energy [28], Power Ledger [29] and Grid Singularity [30]. These are the most similar approaches we have found regarding our work. However, their proprietary nature deprives householders of flexibility in the characteristics and variety of the smart meters to be used and in the possibility of creating new contracts on the part of the users. The absence of these contracts also produces a lack of customization of the monitoring and trading systems and authorization systems integrated in the blockchain infrastructure.

Finally, and in relation to security in the blockchain networks, although blockchain supports non-repudiation and integrity, data privacy and confidentiality are often not preserved. Data content is seen by anyone in the network for verification and mining purposes [31]. Attribute-based encryption (ABE) techniques are used for providing privacy in an IoT ecosystem.

On the use of security credentials for connection with off-chain databases, some works [32] employ the so-called chameleon hash functions to re-write and compress the content of any number of blocks in decentralized services. With this approach, it is possible to remove inappropriate content and the possibility to support applications requiring re-writable storage, to “the right to be forgotten.”

In relation to the transaction information, which can disclose user’s privacy, a node cooperation verification approach is proposed to achieve k-anonymity privacy protection [33]. K-anonymity protection ensures that information for each user contained in transactions cannot be distinguished from at least *k* − 1 users whose information also appears in transactions. In order to maintain existing smart devices and connect them to the blockchain network some authors [34] propose Blockchain Connected Gateways (BC Gateways) to protect users from providing personal data to IoT devices without user consent. In addition, the gateways store user privacy preferences on IoT devices in the blockchain network.

Regarding the management of access permissions, some works contribute to manage privacy policies in blockchain [35]. Specifically, they develop an automated access-control and audit mechanism that enforces users’ data privacy policies when sharing their data across third parties. With this approach data users can automatically apply their privacy policy on data operations and track the flow of that data across various stakeholders.

## 3. System Requirements and Architecture of the Solution

In this section, we propose the needs to be covered through this research work, by formalizing the problems analysed in Section 2 and by focusing on these functionalities through of the proposal of the architecture of our solution.

### 3.1. System Requirements

The paper proposes a blockchain-based authorization system in order to provide optimal resource management in smart communities, as a contribution to the field of CEMS.

As described in the introduction, there is a set of problems associated with energy management in a community of neighbours, in which technological progress has allowed an accurate measurement of individual energy consumption in real time and a set of configurable smart devices allowing to adjust the energy consumption in order to optimize a collective consumption, as a sum of the individual consumptions of the householders.

We present a set of requirements through a case study of energy consumption in a smart community. We consider a smart home owner Bob who wants to increase his energy consumption quota (amount of resources he can consume according to the contract with his community) as he is having visitors during the weekend. First, he needs to know the power demand of his home in real-time, to determine current and future needs (*REQ#1*). To maintain the overall community consumption, we need to establish mechanisms to adapt or distribute the consumption to cover those demands (*REQ#2*).

For Bob to modify his energy consumption, without altering the overall community consumption, an incentive system (*REQ#3*) is required, so that Alice, who is leaving her house during the weekend due to vacation, can obtain some benefits on reducing her consumption capacity in favour of Bob.

With the purpose of registering this exchange for a future analysis or provision of evidences, an infrastructure is needed that keeps the history of the relations maintained by the neighbours of the smart community in relation to the monitoring of their energy consumption, delegation requests for monitoring and resource trading (*REQ#4*).

Finally, to avoid making a profile of the social habits of Bob and Alice protection mechanisms are required (*REQ#5*), preventing energy consumption profiling, as resource consumption belongs to the private sphere and should not be accessible from outside the community. In addition, for the participants in the infrastructure to access to information, mechanisms are required for the management of access privileges (*REQ#6*) on common and private resources, for the delegation of functions of device monitoring and consumption among neighbours.

Finally, in relation to trust, trust provision systems [36] must comply with the requirement that the information introduced by smart devices has not been altered (*REQ#7*) by the agents in charge of entering the information.

### 3.2. Architecture of the Solution

The use of blockchain technology allows us to strengthen the contributions to solve the requirements mentioned in the previous section. We use the smart contract concept [37] which enacts blockchain transactions when certain conditions have been met. The infrastructure verifies the consistency of resource consumption and the enactment of trading agreements.

**Figure 1:**
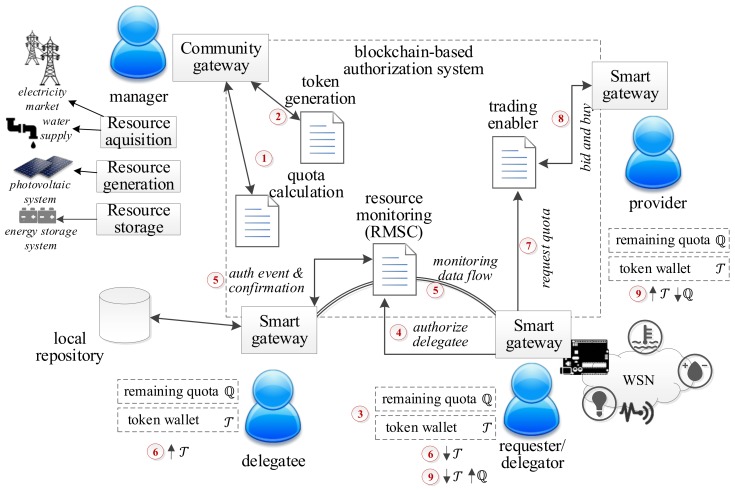
Architecture of the blockchain-based authorization system.

Figure 1 presents an architecture of the solution. The smart community performs the functions of resource acquisition, generation and storage and communicates to the rest of the system by the Community gateway. The smart home integrates a Wireless Sensor Network (WSN) which generates information of resource monitoring (*REQ#1*). The smart home communicates with the rest of the system by the Smart gateway.

We propose an authorization solution based on blockchain to implement the functions of resource monitoring and trading (*REQ#2*), through the provision of smart contracts: quota calculation, token generation, consumption monitoring and trading enabler.

We have defined the roles and the physical elements involved in the processes of delegation of monitoring (4–6) and the process of trading to request the ability to consume resources initially associated with another user, which we call quota in this work (7–9).

Initially the community manager performs the processes of quota calculation (1) and token generation and distribution (2) through the Community gateway. In Section 5.1 we propose a consumption model for smart communities and we will explain the novelty of the use of blockchain tokenization mechanisms for the provision of incentives (*REQ#3*). We consider for now that each user representing a smart home has been provided with a quota of ℚ consumption depending on the contracted rate or the agreement taken within the community. This user also has a limited number of virtual currencies or also called tokens, stored in a token wallet, with which the user can negotiate to increase or decrease his/her quota limit (3).

We also consider that the consumption monitoring contract is monitoring and collecting the consumption values of each smart home. Thanks to this infrastructure, only the authorized users (provided with the appropriate key) can incorporate information to the blockchain network (*REQ#5*) and relations maintained by the neighbours are permanently registered (*REQ#4*). However, if some third party is able to introduce a node in the network, Section 4 proposes mechanisms so that participant nodes that have the knowledge about the energy consumption of a smart home, can hide or reveal this information to other nodes, keeping in any case, the trust between the nodes and the correct functioning of the network.

Suppose a delegator user wishes to grant access (*REQ#6*) to another user so that he can visualize the consumption of resources (i.e., he will spend a few days away from home and wants to designate a responsible person, or to keep the execution of an ambient intelligence scenario [38]). The delegator must communicate with the consumption monitoring contract and request an authorization process (4). Once the authorization has been confirmed and the permissions have been received (5), the delegatee is now responsible for the monitoring of the delegator’s environment consumption and the monitoring data flow begins (5). This responsibility is valued in the form of tokens, which will be transferred from the delegator’s account to delegatee’s account (6). Local repositories are defined for each smart home so that only the repository owner and the authorized persons can access repository information.

If the user considers that he needs to increase his quota of consumption of resources because he plans to face a greater consumption (either total consumption or instantaneous power) in a certain time horizon, he can consume the functionality of the trading enabler contract (7) that will put in contact the quota requester with another user that will act as quota provider. The trading process maintains additional problems that will be dealt with in Section 5.3, where an auction-based trading model is proposed as a contribution of this work, which will be compared quantitatively with other models. Once the process (8) has been solved, the user that has accepted the request (quota provider) exchanges part of its quota for a value expressed in tokens (9).

One of the novelties of designing our authorization system using this technological support is the maintenance of the reliability of the information (*REQ#7*). Information is distributed among different nodes as chained blocks. Each block stores data and metadata information, which can also describe an application or service. Blocks are chained by signatures (hash functions), protecting the stored content. Any unauthorized change is detected by signature mismatch and any posterior block considering this anomaly gets also invalid.

## 4. An Authorization System for Trustworthy Resource Monitoring

To enable the quota and resource management (processes (1), (6) and (9) in Figure 1) in the proposed architecture, each smart home must monitor its resources using the corresponding WSN (see Figure 1). Sensor nodes in that WSN are physically connected to the Smart gateway (usually through wireless communication links), so data generated by these sensor nodes are necessary collected by the Smart gateway, which acts as concentrator for this WSN. However, the Smart gateway is also the controller of the smart home, responsible of communicating with the home inhabitants (using mobile application, for example), pre-processing data for detecting alarm situations, communicating monitoring information to the central management infrastructure (Community gateway) and receiving events and alerts from the Community gateway (where resource quotas are controlled).

Two important characteristics must be considered about these controller functions. First, transactions generated and stored in the blockchain infrastructure (processes (7) and (8) in Figure 1) contain private information which must be protected from unauthorized accesses. Thus, users’ personal information will be stored in local repositories, only accessible by the corresponding local Smart gateway (see Figure 1); and will never be transmitted or communicated. Besides, monitoring information (which must be shared among entities in the system) must be encrypted before being transmitted or stored (processes (7) and (8) in Figure 1), as it refers to critical infrastructures. And, second, the proposed solution to be useful, all members in the smart community should trust the information provided by each individual smart home. To make trustworthy all these transactions, they will be performed through the blockchain network.

In this context and considering previous requirements *REQ#6* (management of access privileges) and *REQ#7* (information has not been altered by the agents in charge of entering the information), any proposed secure monitoring solution must enable three basic situations: the secure data storage in the blockchain network, the universal access to data by the Community gateway and the delegation of controller functions among Smart gateways. This section describes this technology. 

### 4.1. Trustworthy Secure Monitoring Solution

**Figure 2:**
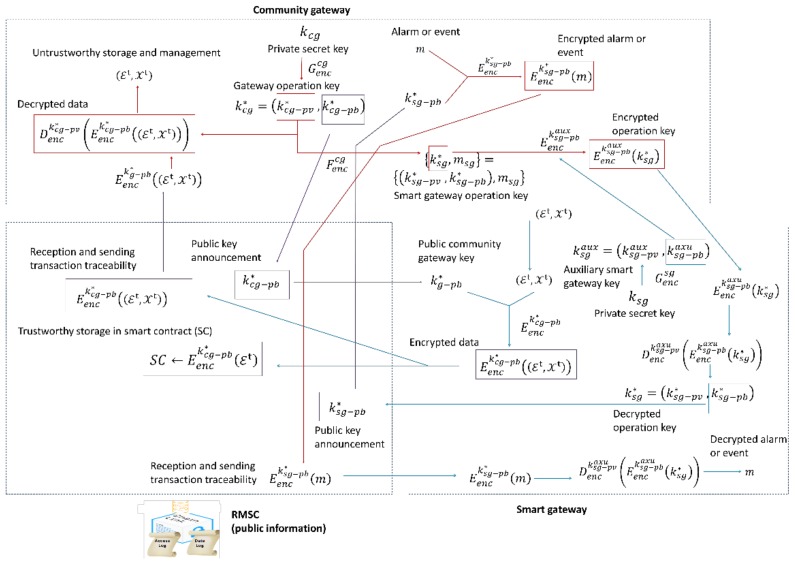
Architecture for the trustworthy secure resource monitoring solution.

In the proposed trustworthy secure monitoring solution (see Figure 2) three basic entities are distinguished: Smart gateway, Resource Monitoring Smart Contract (RMSC) and Community gateway.

The Smart gateway collects a set $D^{t}=\;\left\{d_{1}^{t},\ldots ,d_{N}^{t}\right\}$ of data from the WSN, each T seconds. N is the cardinality of the dataset and each particular datum $d_{i}^{t}$ has a timestamp t. From this data set $D^{t}$, the Smart gateway, using a processing vector function $f_{p}$, calculates two new datasets: the set of essential data $E^{t}=\;\left\{e_{1}^{t},\ldots ,e_{M_{1}}^{t}\right\}$ and the set of extended data $X^{t}=\;\left\{x_{1}^{t},\ldots ,x_{M_{2}}^{t}\right\}$ as shown in (1).

(1) $$\;\left(E^{t},\;X^{t}\right)=\;f_{p}\;\left(D^{t}\right)\;$$

The set of essential data is employed to perform the basic quota control and the extended dataset is employed to execute enhanced management algorithms. Both data sets must be sent to the Community gateway in a secure manner (as presented in process (5), Figure 1, although between Smart gateway and Community gateway), for which it is employed an asymmetric key encryption scheme (see Figure 2).

A public key encryption (PKE) scheme is a 3-tuple $\left(G_{enc}^{cg},E_{enc},D_{enc}\right)$. The first element is a key generator, the second one is the encryption function and the third one is the decryption procedure. From this PKE, we only require to be homomorphic (this property will be extensively employed in Section 4.2). As can be seen, using the key generator, the private secret Community gateway key $k_{cg}$ and a n-tuple of additional parameters to increase the generator entropy, a gateway operation key $k_{cg}^{*}$ is constructed. This operation key is a 2-tuple, including (as any PKE) two different keys: a private gateway operation key $k_{cg-pv}^{*}$ and a public gateway operation key $k_{cg-pb}^{*}$ as shown in Equation (2).

(2) $$\;G_{enc}^{cg}\left(k_{cg},\left\{p_{1},\ldots ,p_{n}\right\}\right)=k_{cg}^{*}=\left(k_{cg-pv}^{*},\;k_{cg-pb}^{*}\right)\;$$

The Community gateway announces and publishes the public Community gateway operation key $k_{cg-pb}^{*}$ through the blockchain network. All Smart gateways in the private network will receive an event about this publication and they will be able to read that public key using the getter functions in the RMSC. By means of this mechanism, the operation is trustworthy (thanks to the trust provision property of blockchain networks) and prevents some cyberattacks such as Man-in-the-Middle.

Each Smart gateway, using the published public key $k_{cg-pb}^{*}$ and the encryption algorithm $E_{enc}$ protects and encrypts both datasets $\left(E^{t},\;X^{t}\right)$. Although, at this point, the encrypted information could be sent through a common and unsecure communication channel, in this trustworthy solution, this sending is made through the blockchain network (as in the Community gateway public key publication).

This encrypted information $E_{enc}^{k_{cg-pb}^{*}}\left(\left(E^{t},\;X^{t}\right)\right)$ is redirected to the Community gateway by the RMSC. However, with this transaction, it is automatically created a trustworthy traceability record in the blockchain network, describing all the performed transactions. These transaction traceability solutions, nevertheless, cannot be easily auditable (as they are based on the calculation of hash functions); thus, to address this problem, the RMSC also stores the encrypted essential dataset $E_{enc}^{k_{cg-pb}^{*}}\left(E^{t}\right)$. The RMSC stores the newest information and, if configured, a historical record of the last L essential datasets, as shown in Equation (3). This information makes up a trustworthy data repository which will be revealed if a conflict arises. As the information is encrypted, although data stored in a blockchain network is publicly available, no unauthorized entity could read the content.

(3) $$\;SC\leftarrow \left\{E_{enc}^{k_{cg-pb}^{*}}\left(E^{t_{0}}\right),\ldots ,E_{enc}^{k_{cg-pb}^{*}}\left(E^{t_{L}}\right)\right\}\;$$

Using the corresponding private key $k_{cg-pv}^{*}$ and the decryption algorithm $D_{enc}$, the Community gateway may extract the original datasets, which feed the management and quota control algorithms and will be stored in a global data repository. Using these same elements, the trustworthy data repository in the blockchain network may be decrypted and exposed by the Community gateway if necessary.

On the other hand, using all received information, management and control algorithms in the Community gateway generate alarms or events to inform the Smart gateways about relevant situations. To also maintain privacy about this information flow, an additional mechanism is considered.

Each Smart gateway, using a private key $k_{sg}$ and a new key generator $G_{enc}^{sg}$, calculates an auxiliary Smart gateway key $k_{sg}^{aux}=\left(k_{sg-pv}^{aux},\;k_{sg-pb}^{aux}\right)$, for an auxiliary PKE $\left(G_{enc}^{sg},\;E_{enc},D_{enc}\right)$, whose structure is equivalent to which described above.

Through an auxiliary communication channel (not through the blockchain network) the Smart gateways communicates the corresponding public $k_{sg-pb}^{aux}$ key to the Community gateway. Blockchain is a technology to make agreements about certain information among a community of users. In this proposal, public operation keys are published and transmitted through blockchain networks to turn them into trustworthy information. However, when only two entities must perform a private negotiation process (such as in the exchange of auxiliary keys), blockchain networks are useless (there is not a community of users to support the system) and, besides, private information is publicly communicated and stored. Thus, blockchain networks must only manage public information to be shared among all members in the community. Definitely, as, in this case, an agreement and trust among all the gateways in the private network is not required, keys do not have to be exchanged by means of the blockchain. In parallel, the Community gateway employs a new key generator $F_{enc}^{cg}$ that presents the vector structure of Equation (4). It receives as parameters the private operation key $k_{cg-pv}^{*}$ and a n-tuple of additional parameters to increase the generator entropy. As result, the generator produces the Smart gateway operation key and a piece of information $m_{sg}$, which is unique for Smart gateway and is called “homomorphic constant.”

(4) $$\;F_{enc}^{cg}\;\left(k_{cg-pv}^{*},\left\{r_{1},\ldots ,r_{n}\right\}\right)=\left\{k_{sg}^{*},\;m_{sg}\right\}=\left\{\left(k_{sg-pv}^{*},\;k_{sg-pb}^{*}\right),m_{sg}\right\}\;$$

This homomorphic constant has no use in this Section, although will be essential for the delegation process described in Section 4.2. The Community gateway, then, using the received auxiliary public key $k_{sg-pb}^{aux}$ encrypts the new Smart gateway operation key $k_{sg}^{*}$ for the Smart gateway which is sent in return to the Smart gateway through the auxiliary communication channel. The Smart gateway, then, employs the auxiliary private key $k_{sg-pv}^{aux}$ to extract the operation key $k_{sg}^{*}$ and publishes the corresponding public key $k_{sg-pb}^{*}$ through the blockchain network to make this operation trustworthy.

Any gateway in the system, including the Community gateway, can (at this point) obtain this Smart gateway operation public key $k_{sg-pb}^{*}$. The Community gateway obtains it and uses the encryption algorithm to encrypt the alarm or event m, to be sent to the Smart gateway. The resulting encrypted event $E_{enc}^{k_{sg-pb}^{*}}\left(m\right)$ is published in the blockchain network from where all gateways could retrieve it. However, only the Smart gateway to which the event is referred has the appropriate operation key $k_{sg-pv}^{*}$ to apply the decryption algorithm and obtain the clear event information m. The Smart gateway can, then, alert the users using the personal information it maintains and only they can read the event information.

The proposed solution ensures the privacy and trustworthiness of all transactions and the monitoring information.

### 4.2. Secure Controller Functions Delegation

**Figure 3:**
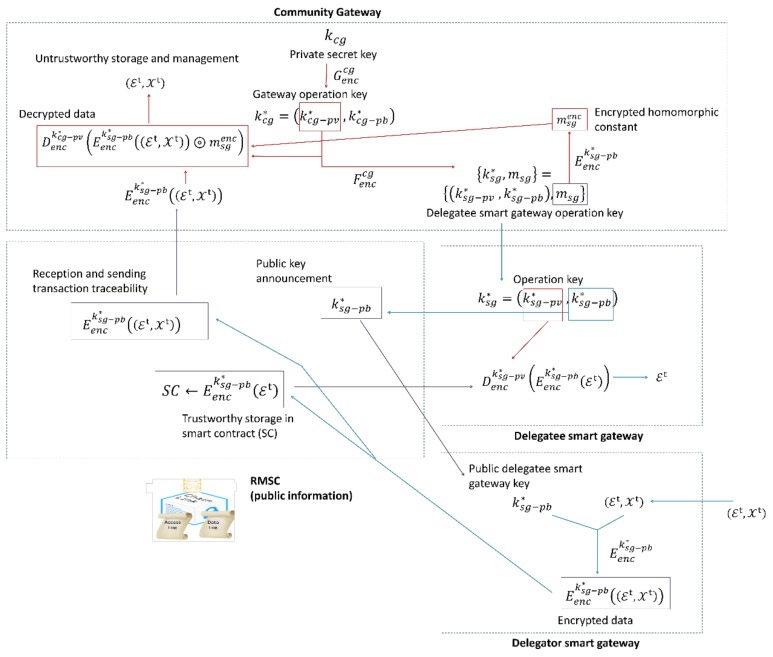
Architecture for the secure controller functions delegation.

The proposed solution in Section 4.1 enables two functionalities, the secure data storage in the blockchain network and the universal access to data by the Community gateway. In this section, a procedure to allow the delegation of controller functions between Smart gateways, guaranteeing also the secure data storage in the blockchain network and the universal access to data by the Community gateway is described (see Figure 3).

Although no Smart gateway can delegate its functionalities as WSN concentrator (because sensor nodes are physically connected to it), user may ask these devices to delegate their controller function to other Smart gateway. As users’ personal information is never shared, if one inhabitant desires other user to take care of his smart home (receiving alerts, etc.), the corresponding (delegator) Smart gateways (in the original smart home) must delegate its controller functions in the other (target, delegatee) Smart gateway.

In the proposed trustworthy delegation solution (see Figure 3) four basic entities are distinguished: the delegator Smart gateway, the delegatee Smart gateway, the RMSC and the Community gateway.

As said in Section 4.1, for each Smart gateway the Community gateway calculates an operation key $\left\{k_{sg}^{*},\;m_{sg}\right\}=\left\{\left(k_{sg-pv}^{*},\;k_{sg-pb}^{*}\right),m_{sg}\right\}$, which includes also a homomorphic constant $m_{sg}$. This key is exchanged with Smart gateways in a secure manner using an auxiliary key and auxiliary communication channel.

When a delegator gateway decides to delegate its functionalities, it starts a negotiation procedure with the delegatee gateway, including the capabilities of both elements [39]. Both entities must accept the delegation before any change in the trustworthy monitoring solution. Defining this negotiation process is not the objective of this paper and many examples may be found in the literature [39], so the proposed solution considers all involved elements have accepted the final delegation.

To execute a delegation process, the delegator Smart gateway obtains from the RMSC in the blockchain network the operation public key of the delegatee Smart gateway $k_{sg-pb}^{*}$. Then, using this key and the encryption algorithm the delegator gateway encrypts the monitoring information datasets $\left(E^{t},\;X^{t}\right)$. The RMSC receives both encrypted datasets and operates as described in the previous section (they are redirected to the Community gateway and the essential set is also stored). In that way, the delegatee Smart gateway may recover the essential monitoring information from the encrypted set $E_{enc}^{k_{sg-pb}^{*}}\left(E^{t}\right)$, using its operational private key $k_{sg-pv}^{*}$. With this information, the delegatee gateway may perform the local control functions of the delegator gateway.

At the same time, the delegator Smart gateway informs the Community gateway about the delegation decision. Then, all events and alarms about the delegator smart home will be encrypted by the Community gateway using the delegatee Smart gateway public key $k_{sg-pb}^{*}$, what will enable it to decrypt and read the corresponding alarms and events.

As can be deducted from the described procedure, delegation permission is a temporary permission. While delegation permissions are valid, delegator Smart gateway has no access to alarms or events generated by the Community gateway (as they are managed by delegatee Smart gateway). However, it still has access to information generated by the Wireless Sensor Network it belongs to (as said, these functionalities cannot be delegated). Moreover, although this procedure is not described in this paper, it is also possible to cancel the delegation permissions. In order to do that, delegator Smart gateway must send a message to the Community gateway indicating the end of the granted delegation permissions and also must notify to the delegatee Smart gateway the end of the delegation process. Then, Smart gateways and Community gateways return to a standard working scheme, as described in Section 4.1 and Figure 2.

With this scheme, Smart gateways may delegate controller functions and stored data in the blockchain network remains private and secure. However, it is still necessary to guarantee the global access to data of the Community gateway. To enable this functionality, the Community gateway recovers the homomorphic constant associated to the delegatee Smart gateway $m_{sg}$.

It must be, now, considered that the proposed PKE must fulfil a relation between the Community gateway public key, Smart gateway public key and the homomorphic constant. In this case we are considering the relation for the delegatee Smart gateway in Equation (5). Although the constant $m_{sg}$ is very difficult to calculate, some works have already proved its existence [40].

(5) $$\;E_{enc}^{k_{cg-pb}^{*}}\;\left(m\right)=E_{enc}^{k_{sg-pb}^{*}}\;\left(m_{sg}⊛m\right)\;$$

being ⊛ an algebraic operation.

At this point and considering that the selected encryption scheme is homomorphic, it is possible to rewrite the encryption of message m in terms of a new parameter that is unique for each Smart gateway, $m_{sg}^{enc}$; and that corresponds to the encrypted homomorphic constant using the Smart gateway operation private key, as shown in (6).

(6) $$\;E_{enc}^{k_{cg-pb}^{*}}\;\left(m\right)=E_{enc}^{k_{sg-pb}^{*}}\;\left(m_{d}⊛m\right)=E_{enc}^{k_{sg-pb}^{*}}\;\left(m_{d}\right)\;⊚\;E_{enc}^{k_{sg-pb}^{*}}\;\left(m\right)==\;m_{sg}^{enc}⊚\;E_{enc}^{k_{sg-pb}^{*}}\;\left(m\right)\;$$

being ⊛, ⊚ algebraic operations.

In this expression, $m_{d}$ is a specific homeomorphic constant for the selected encryption scheme and the employed Community gateway and Smart gateway operation keys. This constant must be calculated each time a delegatee Smart gateway operation key is generated.

Then, in the Community gateway and using the properties of homomorphic encryption, the monitoring information datasets are obtained using the Community gateway private key, though the use of the encrypted constant $m_{sg}^{enc}$ shown in Equation (7).

(7) $$\;D_{enc}^{k_{cg-pv}^{*}}\left(E_{enc}^{k_{cg-pb}^{*}}\;\left(m\right)\right)=\;D_{enc}^{k_{cg-pv}^{*}}\left(m_{sg}^{enc}⊚\;E_{enc}^{k_{sg-pb}^{*}}\;\left(m\right)\right)\;$$

Thus, the global and secure access to data of the Community gateway is also guaranteed, even in a delegation situation.

## 5. Trustworthy Resource Trading Proposal

Once the theoretical model for the authorization between users and towards the communications infrastructure is described, we describe the contribution of trustworthy resource trading. In this section, we talk about the provision of a token exclusively designed for operations within the smart community, which we call Smart Community Token (SCT) through a blockchain smart contract. We propose a resource consumption model based on the interdependence of consumption variables and tokens. After that, we describe the functionalities available in the infrastructure for the management of this asset, including the support for PoW and PoA consensus mechanisms. We will also introduce a new trading model, specific for exchanges of resources by cryptocurrencies, since it allows us to take advantage of the automatic generation of cryptocurrencies in the network and the theory of games in the auction stage.

### 5.1. Resource Consumption Model

In relation to the devices present in Wireless Sensor Networks, in general, it is known the average and maximum consumption of the composing devices, which is conditioned to multiple factors [41] such as the type of operation (continuous or periodic) and mode of consumption (programmable, manual, or unknown) and the dependency between devices (independent, with dependency between devices and with dependence on user actions).

The resources that we consider in this work that affect a smart home can be classified as average power, maximum instantaneous power and average water consumption. This classification is simple but a useful way to model a home monitoring environment, adapting it to different levels of demand and considering two types of resources, water and electricity, better evaluating the consumption of some sensors such as irrigation valves or water pumps.

We also consider that we can measure the consumption generated in a house, by checking the electronic control switch (norm UNE 20.317 in Spain). This standard regulates the excesses in contracted power when maximum current values are reached, proceeding to report breaches of contract or cuts in supply at times inversely proportional to the difference between the consumed and the nominal power. A smart power meter can implement these standard functions and anticipate situations of breach of contract.

Considering $P_{Hi}$ the contribution of household *i* to the general consumption, we associate an economic compensation $M\left(P_{Hi}\right)$ to the assignment of instantaneous power. This compensation measured in number of tokens T is reflected in Equation (8)

(8) $$\;M\left(P_{Hi}\right)=\;T\cdot Z_{T}\cdot W_{P}\left(t\right)\;\;$$

Being $Z_{T}$ the price of the token in the cryptocurrency of the blockchain network used and $W_{P}\left(t\right)$ a weight that depends on time and that allows to make the economic compensation more or less attractive in an auction stage.

In addition to minimizing the power, another aspect to be taken into account is the energy consumption limit assigned to the set of addresses $E_{c}$ that must be met by Equation (9) for each house, such as *P_Hi_ ∗ t*, where *t* is the billing period.

(9) $$\;E_{c}\geq \overset{n}{\underset{i}{\sum }}E_{Hi}=\;\overset{n}{\underset{i}{\sum }}\int P_{Hi}dt\;\;$$

Being $E_{Hi}$ the energy consumed in a smart home during its billing period and therefore the integral of the power consumed during that period. That energy consumed must be within a set limit $E_{ci}$ being $E_{c}=\overset{n}{\underset{i}{\sum }}E_{ci}$ different for each home, depending on the contribution made to the community contract.

We associate an economic compensation $M\left(E_{Hi}\right)$ to the trading of the energy limit. This compensation measured in number of tokens T is reflected in Equation (10)

(10) $$\;M\left(E_{Hi}\right)=\;T\cdot Z_{T}\cdot W_{E}\left(t\right)\;$$

Being $Z_{T}$ the price of the token in the cryptocurrency of the blockchain network used and $W_{E}\left(t\right)$ a weight that depends on time and that allows to make the economic compensation more or less attractive in an auction stage.

In the case of water consumption in an address, having a similar characteristic to energy consumption, we avoid duplicating the mathematical definitions for a more comfortable reading of the work, considering only that there is another economic compensation $M\left(W_{Hi}\right)$ to the assignment of the limit of water consumed. This compensation measured in number of tokens T is reflected in Equation (11)

(11) $$\;M\left(W_{Hi}\right)=\;T\cdot Z_{T}\cdot W_{W}\left(t\right)\;$$

Being $W_{W}\left(t\right)$ a weight that depends on time and that allows to make the economic compensation more or less attractive in an auction stage.

### 5.2. Smart Community Token Definition and Provision

Blockchain tokenization functionality allow the establishment of a token, or tradable asset unit enabling resource trading in a peer-to-peer network without a central price signal. In a conventional power grid, energy prices are determined by a central authority. In a smart community, agents should be allowed to negotiate resource prices, hence creating a dynamic market of resource trade. Such a market-based energy trade decreases dependency of agents on a central energy provider, as energy supply and demand are matched directly between individual agents, resulting in a more decentralized and competitive environment. Our Smart Community Token satisfies this requirement and enables peers to anonymously negotiate energy price and securely perform trading transactions.

SCT represents the amount of resources to be produced or consumed (for example, power, measured in watts), the time interval in which energy is to be produced (or consumed). These tokens are distributed among household owners by the smart community, which also validates that the specific seller has the energy capacity for feasible trades given the assets. Once participating accounts have these assets into their blockchain wallets they can trade between themselves using their anonymized addresses, hiding their identity.

**Figure 4:**
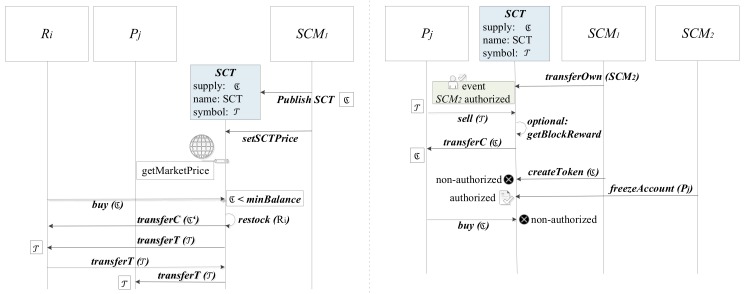
Token provision through Smart Community Token contract.

The SCT is defined as a smart contract in the blockchain infrastructure. Therefore, the properties it has are equivalent to the set of functions defined in this contract. Figure 4 describes the characteristics of the defined token and its provision on the infrastructure proposed in this work.

We consider a case in which we have a set of Smart Community Managers $\left\{SCM_{1},SCM_{2}\right\}$ and two sets of smart gateways $R_{i}\;:i=\left\{1\;....\;m\right\}$ $P_{j}\;:j=\left\{1\;....\;n\right\}$ acting as requesters and providers of resource consumption quota. We can also call the requests and providers as buyers and sellers, since they will purchase and sell resources respectively. In Figure 4 (left) we can see how the SCT contract is deployed by the $SCM_{1}$ through the *Publish SCT* order, through which an initial token supply, a name and a symbol are provided to differentiate this currency.

We differentiate the terms *currencyAmount* (ℭ) and *tokenAmount* (T) as the amount of economic assets in the form of the natural cryptocurrency of the implementation of the blockchain network that we are using (for example Ether, in the case of an implementation of Ethereum) and the number of SCT tokens that have been created for the exchange within the smart community. As each node can exchange tokens for currency by using this contract, it is important that enough currency is included into the contract so it can use this currency to buy back all the tokens on the network if all users were to perform this exchange at the same time.

The exchange of *currencyAmount* (ℭ) and *tokenAmount* (T) is regulated by the function *setSCTPrice*: SCM→SCT with the parameters *buyPrice* and *sellPrice*, being able to differentiate a different price for purchases (change ℭ for T) and sales (change T for ℭ). We want the value of the token to fluctuate the same way as the price of the resource in the common market and not to be penalized by the price of ℭ. Therefore, we determine the $Z_{T}$ of Equations (3) and (5), defined as the price of the token in the cryptocurrency of the blockchain network. Since this value is a constant floating price, we provide a trusted data feed [42] connected to the smart contract that periodically checks the price of the cryptocurrency (*getMarketPrice*) in relation to the price of the resources offered to the smart community by the resource provider.

The first operation to be carried out is the provision of tokens to the users of the network. We consider that users have ℭ, the official cryptocurrency of the network. Using the *buy*: $R_{i}\to SCT$ function with the *currencyAmount* parameter in ℭ. The reception of this invocation in the SCT contract will result in the execution of *transferT*: $SCT\to R_{i}$ function, with the parameter *tokenAmount* in T. By invoking *buy*, users send value expressed in their cryptocurrency and receive tokens in exchange. Similarly, the inverse operation *sell*: $P_{j}\to SCT$ with the *tokenAmount* parameter, will execute the *transferC*: $SCT\to P_{j}$ function with the *currencyAmount* parameter.

Automatic token exchange mechanisms are established by cryptocurrency when the network suspects that a user may not have enough currency to pay the necessary commissions to interact with the contract. Commissions, or service fees, are inherent to blockchain, as prevent abuse in transactions that could overload the network. Commissions are calculated based on the amount of effort required to process a given transactions and their cost can be configured by network administrator.

As can be seen, when performing the buy operation, the SCT contract checks if the amount of ℭ that $R_{i}$ has is lower than a *minBalance* limit. If so, the internal *restock* ($R_{i}$) function is executed, which transforms part of the token supply T destined for $R_{i}$ in cryptocurrency ℭ’ and sends it to $R_{i}$ through the *transferC*: $SCT\to R_{i}$ function.

Assuming that $R_{i}$ already has T, if you need to request a quota increase, to consume additional resources, you will invoke the *transferT*: $R_{i}\to \;SCT$ function, providing a value in T and the provider user address of resources $P_{j}$ to make the transfer. This function will check that enough tokens are available and there are no overflows, it will update the accounts and notify the transfer by issuing the *transferT:* $SCT\to P_{j}$ command with the value in T.

In the right half of Figure 4 we can see other functions of the token defined in this work. The contract that governs the operation of the token has the ability to transfer the authorship of the contract. The *transferOwn*: SCM→SCT function requires the provision of the *newOwner* parameter and it can only be invoked by the current owner of the contract. This function allows to define a new owner other than the user who initially deployed the contract. In this way, we make sure that the management of tokens is not lost with changes in the manager of the smart community. Once the invocation of this function is received, the SCT generates an *authorized* event informing all network participants of the change of token manager.

The token manager also has a function reserved to make this asset compatible with the proof of work (PoW) systems. In this way, we define the internal function *getBlockReward* which increases the balance of the miner who mined the block in *rewardValue* tokens. In a PoW environment this function will be executed at the end of the execution of each function of the SCT contract (in the figure it is executed after a call from $P_{j}$ to the *sell*(T*)* function, to encourage mining and, thus, the progress of the network.

To generate new tokens, the function *createToken*: $SCM_{owner}\to SCT$ is defined, invoked with the parameters *tokenAmount* and *destination*, which generates a number of tokens equal to *value* and transfers it to *destination*. If the price of the token in the cryptocurrency of the network does not vary with the generation of new tokens (in a balanced economic environment when injecting new tokens its value would be devalued), it is important to provide currency ℭ to the contract so that it can buy back all the tokens on the market.

Finally, we also provide the contract with the ability to freeze accounts. A frozen account using the *freezeAccount*: $SCM_{owner}\to SCT$ function must provide the *destination* and *freezeStatus* parameters, which contain, respectively, the address of the account to be frozen and a Boolean parameter that determines the application of *freeze* or *unfreeze*. A frozen account keeps its stored tokens but cannot issue or receive transfers, as can be seen in the figure with the call from $P_{j}$ to the buy(ℭ) function, which results in an unauthorized response.

In relation to the compatibility of the defined tokens for the various available consensus systems, we have seen that it incorporates reward operations for the PoW system. However, when the network is composed of a relatively low number of nodes, an attacker with 51% of the mining resources takes control of the network. In addition, the power needed to solve computer problems in PoW produces a consumption with a significant impact on the environment. The final goal of our proposal is to optimize the consumption of resources in a smart community, so a solution that artificially increases the consumption of resources is simply a bad choice.

The proof-of-stake (PoS), however, proposes that a node becomes a block validator when it deposits some currency into a validation contract and if a validator is deemed to be malicious the network simply locks this currency amount away. Although this approach solves the problem of the excessive electricity usage of the PoW, it still does not offer the level of control and security required as anyone on the chain can become a validator if they deposit enough ether into the validator contract.

Therefore, this work deals with the concept of proof-of-authority (PoA), where instead of miners racing to find a solution to a difficult problem, authorized signers can at any time at their own discretion create new blocks. We define a PoA protocol to overcome the previous limitations by extending the 32-byte extra-data of each generated block to 65 bytes, in order to store the miner’s signature. Once a client obtains a block, it matches the miner’s signature contained in the extra-data field to a dynamic list of authorized (trusted) signers.

The list of authorized signers is updated by a voting protocol. Authorized signers can vote for other signers to remain or be kicked of the list. Voting is done by introducing the miner signature in the “nonce” field and 0xf or 0 × 0 to vote in favour of adding or kicking out respectively.

To avoid a malicious signer to discover many blocks in a row, a limitation is imposed so that the signer can sign at most one block of k consecutive blocks being k = Signer_count/2+1, being Signer_count the number of authorized signers valid at a particular instance in the chain.

### 5.3. Resource Trading

We analysed several auction proposals and propose one that fits with the concepts of energy, power and water consumption, considering the requester generally initiating the action and the provider being notified.

Three phases are detected in this auction. The determination phase, the negotiation phase and the payment and resource delivery phase. In the determination phase, a publication-subscription mechanism is used to determine which users are interested in participating in a resource trading process. A list of subscribers with a set of preferences is created, so that only if these conditions are met they will they be notified when there are requests by the other participants.

**Figure 5:**
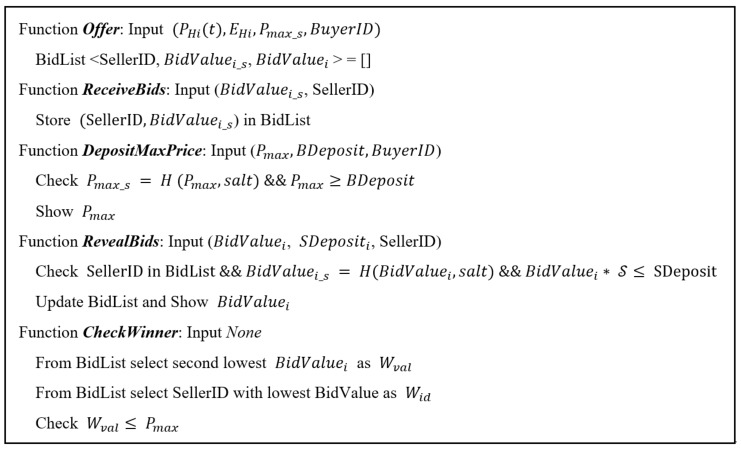
Negotiation algorithm for resource trading.

In the negotiation phase, users use a second-price sealed-bid auction model, also called Vickrey auction [43], adapted to the circumstances of the blockchain network. In this type of auction, bidders submit simultaneous sealed bids to the sellers; the highest bidder wins the object and pays the value of the second-highest bid. In case of blockchain, sealed bids are implemented by signature schemes as explained in the next paragraphs. The negotiation phase can be represented in the algorithm of Figure 5.

The sequential execution of the functions described in the algorithm is performed. First the buyer executes the *offer* function including his resource needs ($P_{Hi}\left(t\right),E_{Hi},W_{Hi}$) and a hash of the maximum price he/she is willing to pay for this lot $P_{max\_s}$.

Our proposed auction requires sealed bids so that no bidders can condition their bids. We also add the maximum price the buy is willing to pay. It must be also sealed. This is important to ensure that bidders value the resource as they think is worth and they do not worry about what other buyers will bid or how much the buyer is willing to pay. Because blockchain transactions are public, bidders are able to see all algorithm inputs. Thus, we are securing sensible information with hashes such as SHA-3. In this way, $P_{max\_s}\;=\;H\;\left(P_{max},\;salt\right)\;$corresponds to a hash function of the maximum buying price in plain text $P_{max}$ with a salt function to make the hashing more robust.

The *ReceiveBids* function receives from each of the vendors their sales price information through the hash function and stores them in a list of sellers. The *DepositMaxPrice* function must be executed by the buyer to deposit its maximum sale price and reveal it to the set of sellers that have made their bid.

The *RevealBids* function allows sellers to formalize their bid by establishing a deposit that guarantees the seller commitment in case it wins the auction. To do so, the function verifies that the seller is registered, that his bid $BidValue_{i}$ corresponds to the value registered in the invocation of the *ReceiveBids* function and that the deposit provided is greater than an amount proportional to his bid. The ratio factor S∈[0,1] determines the value of the deposit in relation to the bid.

**Figure 6:**
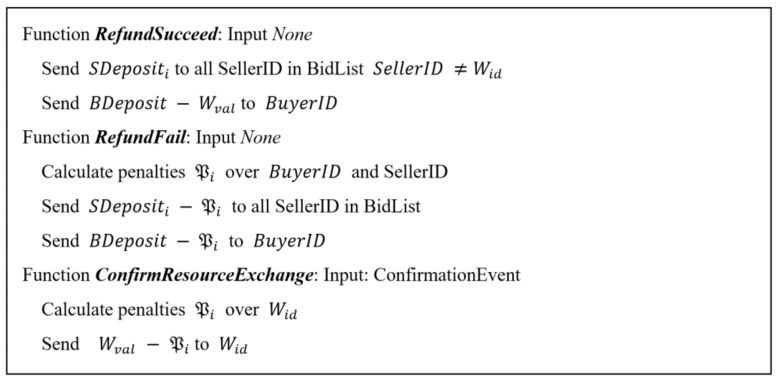
Resource trading, third phase: Payment and resource delivery algorithm.

The *CheckWinner* function selects the seller who made the lowest bid as winner $W_{id}$ and the second lowest bid as purchase value $W_{val}$, following the Vickrey auction model [43]. The function checks that the purchase value is not higher than the maximum purchase value specified by the buyer, giving the auction as terminated and continuing to the payment and resource delivery phase, determined by the operation of the algorithm shown in Figure 6.

Depending on the result of the negotiation phase, if the auction has finished satisfactorily, the *RefundSucceed* function is executed. On the other hand, if it has ended unfavourably, *RefundFail* is executed.

The *RefundSucceed* function returns the deposits to the sellers and the amount of T not spent to the buyer. The *RefundFail* function defines and executes a set of penalties to avoid negotiations that tend to dead-ends, either due to the definition of complex offers or with little interest (high price) that do not gather enough people initially interested in the bid, or due to the establishment of too low or too high price limits. These penalties are deducted from deposits or notified to a general registry.

Finally, for the payment stage to conclude satisfactorily we need to verify that, once the buyer has acquired the resources offered by the seller, these resources have been made available to the buyer. A confirmation event must trigger the execution of the *ConfirmResourceExchange* function in which a penalty for total or partial breach of the agreement is calculated and the payment is made to the seller.

The establishment of $W_{val}$ enables the calculation of $W_{E}\left(t\right)$, $W_{P}\left(t\right)$ and $W_{W}\left(t\right)$, from Equations (3), (5) and (6), weights that depend on time and allow us to do the economic compensation more or less attractive in the auction stage.

## 6. System Implementation

The proposed authorization system for trustworthy resource monitoring and trading has been implemented. In this section, we explain the implementation choices that we selected, such as the blockchain implementation that best adapts to our needs, the technological solutions for the assembly of our proof of concept.

With the aim of creating a smart community for testing, we have implemented four locations, a smart community subsystem (*SC1*) and three smart home subsystems (*SH1*, *SH2* and *SH3*). The locations of smart home in turn had a WSN composed of sensors for the control of water and electricity, in addition to other typical values measured in the WSN such as temperature and humidity. We define $WSN_{SH1}$ as the WSN network for the smart home *SH1*.

### 6.1. WNS for Resource Monitoring

**Figure 7:**
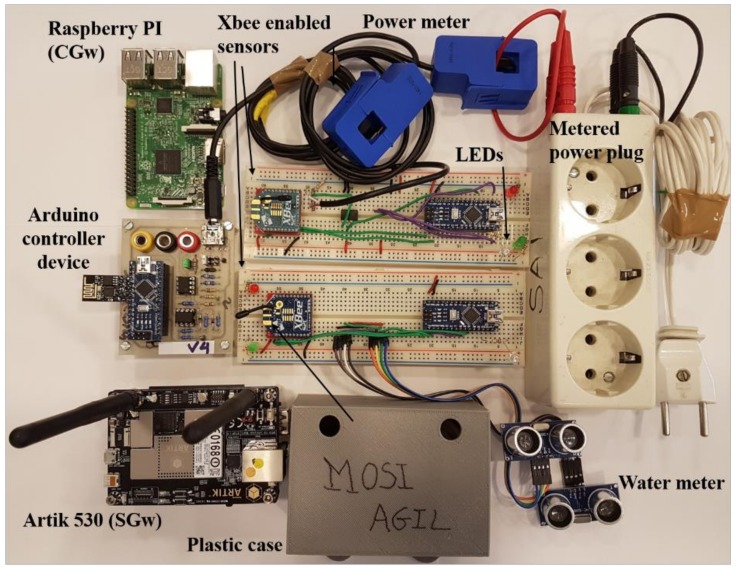
Implementation prototype.

WSNs were implemented using PCB techniques and a regular plastic case. A photo of the prototype is provided in Figure 7.

We use a Raspberry PI 3 model B (Raspberry Pi Foundation, Cambridge, UK) as a Community Gateway (CGw) and the Artik 530 (Samsung, Seoul, Korea) nodes () as Smart Gateway (SGw). We took this decision for the best wireless connectivity features offered by the Artik 530 compared to the Raspberry model, although in terms of the technical performance of the devices, these two are very similar. Being the processor of the Raspberry, a 64-bit quad-core at 1.2 GHz based on BCM2837 and the Artik’s, a 32-bit quad-core at 1.2 GHz based on the ARM Cortex A-9 processor .

Each WSN is composed of a set of sensors and actuators. An Arduino controller device (SparkFun Electronics, Niwot, CO, USA) communicates through a Wi-Fi module with the SGw and that receives consumption information from the power meter that it has connected. We also use other sensors, also based on Arduino Nano, which communicate using the Xbee protocol to the SGw and other connected meters such as the water meter. These devices in turn have different *LEDs* that notify users that the sensor is correctly connected to the WSN and this in turn to the blockchain-based authorization system.

The power meter is a ferrite current transformer connected to a resistive network, which employs induction to generate a voltage proportional to the electricity current going through the transformer’s cable. The sinusoidal signal is rectified, sampled and quantified for later processing.

Water metering module includes two ultrasonic transceivers (sensor HC-SR04, at 40 KHz (SparkFun Electronics, Niwot, CO, USA)), which are situated in both sides of a water pipe and connected to a serial port of the Arduino. For an accurate measurement of the water flow transceivers are activated simultaneously and the procedure known as transit time differential measurement is initiated.

In the controller programming, power and water flow calculation expressions are also corrected by a calibration plan that can be requested by the user to the SGw. In that case devices measure energy and water consumption, comparing them with theoretical values.

Regarding security in communications, the basic PKE in this solution was ElGamal, a homeomorphic encryption scheme based on Diffie-Hellman proposal. As auxiliary encryption schemes to share operation key among Smart gateways and the Community gateway we have selected an algorithm based on Elliptic Curves. We employ 128-bit keys for all the encryption schemes.

### 6.2. Blockchain-Based Authorization and Proof-of-Authority

Each subsystem (*SC1*, *SH1*, *SH2* and *SH3*) is a participant of a private blockchain network, composed of therefore 4 nodes. For the creation of the private network we used the Ethereum implementation provided by the Geth client, version v1.8.13, for Linux/Arm and installed it in 4 Raspberry PI 3 model B. The proposed proof-of-authority (PoA) can be integrated in a private Ethereum network due to the new consensus engine incorporated to Geth 1.8 clients, called *clique*.

We configure our client with the explained PoA mechanism, providing the configuration parameters for PoA support of Table 1. PoA solves the disadvantages of the traditional mechanisms of PoW (high consumption, requires a large number of nodes) or PoS (less control and security level for becoming a validator). After testing numerous combinations of parameters, we have a configuration that allows a stable network with a block generation rate every 10 s, which allows a great immediacy of the negotiation mechanisms that take place in the system.

The *signer_count* and *signer_limit* parameters are very important since they prevent a series of attacks against the PoA in the implemented network that have been checked and whose results are exposed in Section 7. The *block_period* is also a parameter that requires a commitment of performance and consumption. For a transaction to be validated it is recommended to wait a minimum time equivalent to the generation of 7 blocks (7 confirmations, although for maximum security it is suggested up to 12). This means that a block generation time of 1 s required the waiting of 7 s to confirm the transactions. Each generated block has a minimum size of 1024 bytes, so establishing a very high block generation time, like that second would produce 30 GB of data per year, which should be stored by each of the nodes. A block generation time of 10 s would produce a volume of 3 GB; which is more acceptable for a smart city environment. However, a data generation rate of 3 GB per year may not be acceptable in long term. For scalability purposes, a mechanism has been defined that backups the state of the network at a given time and allows to restart the volume of information. The limitation of this mechanisms is that after a state backup the information about each user’s quota would be restarted. This disadvantage is not considered very detrimental since a smart home quota should be renewed monthly or annually at most. Thus, the transaction history might not be necessary after negotiating the next renovation and a digitally signed copy of this history can be stored outside the chain.

### 6.3. Notification Management and User Interaction

A notification system is provided so that users receive in their mobile terminals the events produced in the blockchain network that directly affect their consumption of resources. In each smart home gateway, we use a server in Nodejs, using the Express and Web3 libraries for the creation of a REST interface that can be consumed through HTTPS by a mobile terminal. The Web3 library allows us to register listeners to events issued from smart contracts. In this way, the information generated by the order “*emit*” of the resource trading and token management smart contracts is notified.

We have implemented an application prototype in Android version 6 (API 23) so that users can manage these notifications and in turn invoke calls to the blockchain.

**Figure 8:**
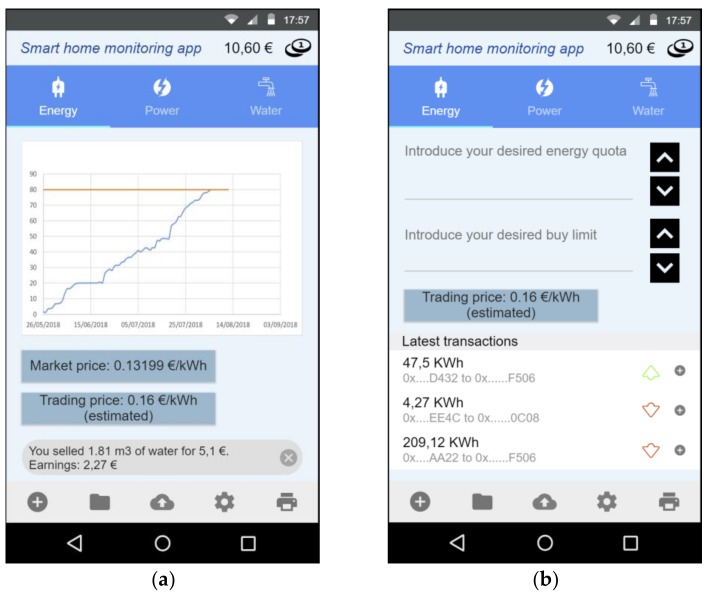
Implementation prototype: (**a**) energy main tab energy; (**b**) transaction tab.

Figure 8 shows two screenshots of the implemented Android application. Figure 8a shows a total balance of tokens, already converted to a physical currency (€) to facilitate the understanding by the inhabitants of a smart home. The application automatically applies a conversion of the value in tokens and the value of the currency of the Ethereum network. Being deployed in a virtual network where the value of the currency does not have to do with the value of the public network, we have adjusted the value of the currency arbitrarily and we have balanced the value of the token (T) to match the value of the quota in tokens with the real currency value of the resource (i.e., € or $).

Three types of resources are considered in the application, Energy, as total energy consumption in kWh, Power, as average power contracted in kW and Water, as volume of water consumed in cubic meters. The mobile application includes a graph about the consumption of these resources and the configured limit from which we are making a consumption higher than the quota contracted and therefore we should proceed with a request for resource trading.

To make this request, the application offers certain information, such as the price of the resource in the common market and the average price at which that resource has been sold in the smart community. This can give the buyer an idea of what he should expect and the purchase limit that he should establish. Finally, this screen offers a history of transactions carried out to inform the status of our wallet.

Figure 8b shows the purchase interest of a certain resource, in this case the *Energy*, where the user must specify the desired energy quota and the buy price limit, which will be used as the maximum price the buyer is willing to pay $P_{max}$, as defined in Section 5.3. This screen also shows information of the estimated trading price, according to latest transactions and a transaction history, indicating if the transactions were over or below this trading price and information on the quota amount and involved accounts.

When a resource request is provided, a mobile notification is shown to other users specifying the requested resource quota value, the market and trading price and some information to help users decide to share their quota, as their surplus energy, or the time that is missing until having more resources available.

## 7. Experiments and Results

The proposed authorization system for trustworthy resource monitoring and trading has been tested in a smart community setting. In this section, we explain the scenarios where we have put into practice the functionality of our system and we have been able to draw some conclusions that determine the usefulness of our proposal and the fulfilment of the scientific contributions raised in this work.

### 7.1. Proof of Authority Suitability and Security Considerations

As we have seen in Section 5.2, this work deals with the concept of proof-of-authority, where instead of miners racing to find a solution to a difficult problem, authorized signers can at any time at their own discretion create new blocks. In this section, we review the security impact and resource consumption of the proposed solution compared to the traditional solution of using PoW.

Our main interest is the protection against the so-called 51% attack, which affects all blockchain networks based on PoW. This attack is based on the design decision that the nodes are usually set up to recognize the blockchain with the most blocks (and therefore the most hashing power) as the correct version of history. The attack consists of using miners with more than 50% of the network hashing power to apply a “double spend” attack, consisting on sending funds to one address on the main chain, while sending the same funds to another address on a forked copy of the blockchain that they are silently mining with more hashing power than the main chain.

In the ‘double spend’ attack, since other nodes only know about the main chain, they will accept this transaction as valid. This malicious node can later release these silently mined blocks and other nodes will accept this as the new “correct chain” since it is longer. This will cause the original transaction to effectively disappear and nodes will recognize the funds as being sent to the address from the new chain instead.

Public blockchain networks that are large enough have enough mining capacity behind them, making it extremely expensive to acquire the necessary hardware to pull an attack like this off. For example, to effectively execute a 51% attack on Ethereum public blockchain an attacker would surpass 249.94 TH/s, its current hashing power at the time of this manuscript preparation.

As a test scenario, we incorporated in our prototype a USB multimeter tester to measure real-time power consumption of a smart node. As a result of our measurements we can see how a fully mining Raspberry PI 3 model B requires 4.9 watts on average (930 mA), which is a low rate. Instead, by using PoA, the smart node consumes 350 mA (1.9 W) of continuous average consumption. These 3 W of difference for each node is not much, so, using the type of low consumption device proposed in our implementation there is not much difference in power consumption between PoW and PoA. However, the use of these resource-constrained devices is a great disadvantage for the PoW model. The resulting measured mining hashing power is 10 H/s which is extremely low rate. Thus, a smart community composed by 40 nodes has only a mining capacity of 0.4 KH/s. An average computer has a computing power of 1 to 10 MHs, in a different order of magnitude. That means that if a high-power computer manages to join the network, without a doubt, it will achieve a 51% attack, allowing a double spend attack and therefore a trust problem.

**Figure 9:**
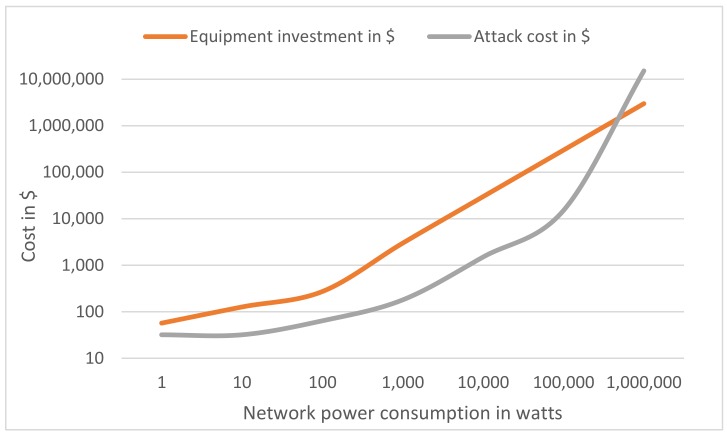
Power consumption and costs for PoW approach.

Using high-power computers, we conducted an analysis comparing the cost of investment in equipment for the smart community, the economic cost of a 51% attack and the impact on power consumption, using as a reference the current best mining ratio available as a consumer solution, using the AMD Radeon RX Vega 64 card, which yields a stock value of 33 MH/s using around 200 watts [44]. We will measure the cost of the attack using a hash renting service called *NiceHash*, with which we can calculate how much it would cost (unitary price $0.91/GH/h) to rent enough hashing power to match the current network hashing power for an hour. The results of this analysis can be seen in Figure 9.

As can be seen in this figure, results indicate that in general, the investment in equipment in order to expand the mining capacity of the network is a bad approach, as 51% attack always requires less investment. Note that the attack does not include the block rewards that the miner will receive for mining. Thus, the difference would be higher.

Regarding the equipment investment, we see that the curve is less pronounced between 1 and 100 W as there is not much variation in the value of the equipment required and that it increases linearly once we reach the best efficiency of W/$, with the equipment described above, which can be used in parallel and in all nodes, which allows scalability while keeping the cost linear.

Regarding the attack cost, we can see that the curve starts in a fixed amount, which are the service fees that we are using and the minimum package to contract. Also, from 100 KW (16.5 THs), the cost of the attack begins to grow exponentially, due to the increase in the prices of hashing services, since it is more difficult for the service to provide such high computing power.

The proof of authority designed in this work allows us to reduce the investment cost, using limited resource devices such as Raspberry PI 3 model B and Samsung Artik 530, not needing a high mining capacity.

Finally, in order to characterize the resilience of the proposed approach against attacks a fault tolerance analysis has been provided. The proposed PoA algorithm belongs to a new family of Byzantine fault-tolerant (BFT) consensus algorithms. Based on BFT theory, a PoA network can tolerate up to ⌈N/2⌉−1 byzantine authority nodes, that is, the network operation will be correct when simple majority of the authority nodes, ⌊N/2⌋+1, are honest. Compared to other BFT algorithms, the design of PoA sacrifices consistency (as signers can mint blocks simultaneously, creating forks that must be resolved by Ethereum GHOST protocol having *eventual consistency*) over availability of information (having faster block generation).

In the configuration proposed in this work, we consider a number of signers *N* = 3, being 4 the number of nodes. As every signer is only allowed to sign once every ⌊N/2⌋+1 blocks, for an attacker to compromise the data on the blockchain, he would have to compromise the whole set of authority nodes. We chose *K* (number of blocks that must elapse so that signer can mint again) to 2. Thus, the proposed PoA mechanism validates BFT theory.

We complement this analysis with the analysis carried out by the team of De Angelis et al. [45], on the behaviour of the PoA clique (on which our proposal is based) by applying the CAP Theorem [46], which states that in a distributed data store only two out of the three following properties can be ensured: Consistency (C), Availability (A) and Partition Tolerance (P). Authors claim that PoA algorithms can give up consistency for availability when considering the presence of Byzantine nodes. In particular, clique-based PoA algorithms are classified as AP (Availability and Partition-tolerance), with eventual consistency guarantees thanks to the Ethereum GHOST protocol. Thus, it is considered that, with this approach, a majority of Byzantine authorities is required to take over the blockchain.

We perform a qualitative analysis on possible security threats on the network to check the trustworthiness level of the network nodes and the confidence of the information circulating in it. This analysis is described in Table 2.

### 7.2. Performance Evaluation for Resource Monitoring

We propose a validation on the performance of our proposal with the aim of verifying if it is feasible to use the authorization system for a correct monitoring and trading of resources in a smart community. For them we consider the variation of the parameters defined in Table 3, where we specify the value used or the range considered for the validation.

We consider the generation factor $G\;=\;\frac{block\_period}{T_{sample}}$ that tells us the number of samples that will be generated in the time in which a block is generated. This factor determines together with $M_{1}$. and *num_SH* if the rate of generation of monitoring information is higher than the rate of registration and storage of information in the infrastructure, and, therefore, the network would tend to saturation. So that this situation does not occur, Equation (12) must be fulfilled:

*num_SH M*_1_ ≤ *G* block_size (12)

We also must take into account an intrinsic restriction of blockchain technology, which offers performance limitations from the computational point of view. The computational cost in the blockchain network is measured through the Gas concept, which is defined as the computational power required to perform an operation. The rates for the execution of transactions and operations are defined in Gas and have their equivalent in the cryptocurrency of the network through the concept of Gas cost. With that, we must take into account the maximum cost that can occur in the creation of a block (and therefore in the execution of all orders that will be stored in that block). That cost is limited by the so-called block gas limit, which in the proposed implementation is approximately 8.0 × 10^6^ gas/block. This cost can limit the execution of transactions that introduce a high volume and information.

**Figure 10:**
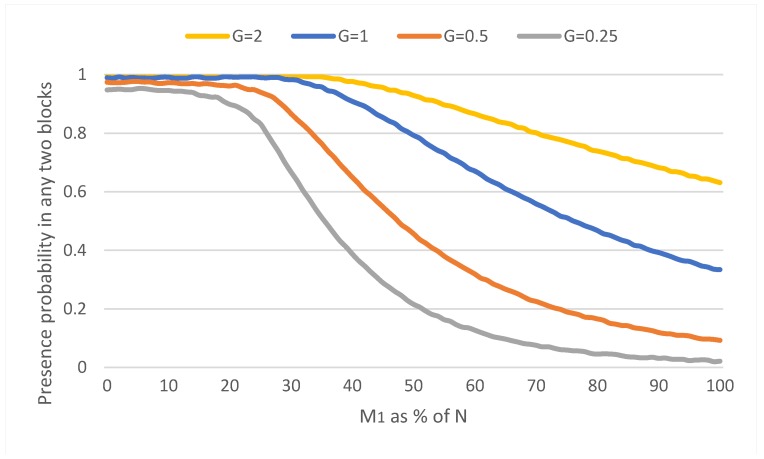
Presence probability of storing $M_{1}$ in next two blocks.

We perform an execution of our system using 40 Docker virtual nodes based on Ubuntu 18.04.1. We measure the average value of the probability that a given transaction is present in the creation of the next two blocks, varying *G* and $M_{1}$. We use the following two blocks and not only the next one because this way we avoid race conditions in the infrastructure, for transactions that occur in a timestamp very similar to the discovery of a new block. The results are shown in Figure 10.

As can be seen, for values of $M_{1}$ very close to *N* = 10 KB, the probability of the presence of said transaction in one of the two consecutive blocks thereof is drastically reduced, following a negative exponential distribution. For example, for a data generation rate of 5 KB of samples (50% of *N*), for the case of generation of 4 samples for each block and 40 smart homes, we see an average probability of only 20% of the transactions being in the next two blocks. This is consistent with the theoretical Gas limit values of the network, which determines the maximum block size (discounting the transaction fee) of 112 KB.

For the tests carried out, we consider that a probability lower than 50% results in a system at the saturation limit and whose performance will not be acceptable.

Also, in relation to the performance of the monitoring and delegation solutions, we performed a test to measure the computational cost of the introduction of information, considering a fixed $M_{1}$ value of 100 Bytes and varying the parameter *L* (number of records of essential data set samples which are simultaneously stored). The blockchain network allows us to measure in a simple way the computational cost of the transactions towards the Smart contracts, since it has been conceived to accurately measure the consumption of resources in the network through the aforementioned Gas concept. For simple operations, the employed Gas is very low (e.g., for addition or subtraction the Gas cost is 3 units), whereas operations managing data are costlier (e.g., loading a word from permanent storage is 200 *Gas*).

In the following table, there is a comparison of the Gas employed by using resource monitoring and delegation solutions (*Smonitoring* and *Sdelegation*) compared to naïve solutions (*Monitoring* and *Delegation*) only relying on the security provided by the blockchain network and, thus, without data privacy (data is stored are read by simple setter and getter functions in the smart contract). Values are provided for each household.

For all these values, in addition, a fixed Gas cost of 32,000 must be added, which is the cost of carrying out each transaction. As can be seen, the computational cost of the proposed solution is about five times greater than the solution without security. It can also be seen how the *Delegation* and *Sdelegation* solutions for the visualization of the information stored in the blockchain are up to 100 times less expensive than solutions based on data writing (*Monitoring* and *Smonitoring*).

We must also take into account again the limitation imposed by the block gas limit, which limits us again the amount of information to be supplied in a single transaction. With a gas limit of approximately 8.0 × 10^6^ gas/block, if some changes should be done in the information stored in the smart contract through the most expensive operations in Table 4, several blocks would need to be generated to complete the operation and thus, more time to validate transactions will be spent.

### 7.3. Validation of Resource Trading

This validation scenario verifies the benefit of the auction system in a simulated environment. We consider a user behaviour model of variable resource expenditure during a month for the case of water. The expenditure model used is based on the household water consumption data offered by the USGS Water Science School [47], such as bath, teeth brushing, hands/face washing, dish washing, drinking water and so forth. The model used is indicative and does not consider a priori consumption habits as schedules, number of people in the houses and so forth. It does consider that the consumption of resources, found in the average values indicated by the reference, occurs at different times. For the behaviour of users to be dynamic, we consider that they select a water saving approach if their consumption is close to the quota limit. In that case they choose over the most resource-saving option, such as taking a shower (10 gallons) instead of a bath (36 gallons), reducing water consumption in dish washing (from 27 to 8 gallons), or using the dishwasher (10 gallons), although there are other consumptions that have a mandatory and fixed fee, such as toilet flushes.

We consider a community formed by 40 homes whose water consumption behaves in this random way. We compare this simulated consumption with the situation of having the resource trading algorithm operative. We simulate the user’s behaviour using the resource trading system so that when they want to consume more than the daily limit, they request that consumption from the home with the lowest consumption level.

**Figure 11:**
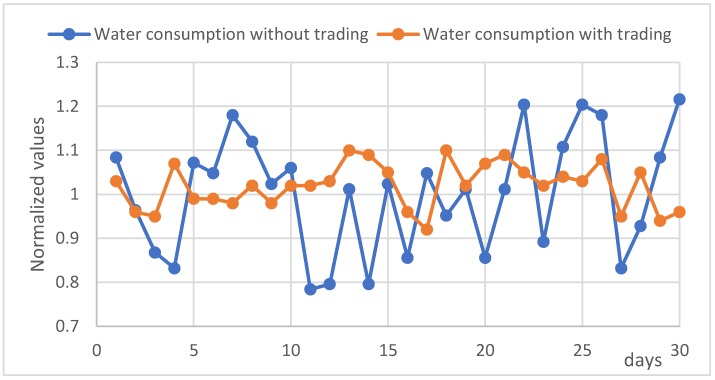
Comparing resource trading with normalized daily values of water consumption.

Figure 11 presents the results of the simulation on a one-month experiment for users using the resource trading functionality proposed in this work and other users not using it.

As we can see the dispersion in the first case is greater, bounded in a range of 22% of the value of the daily consumption quota. As for the excess of average consumption, the consumption measured is a 5.64% greater than the daily limit, in average. In the case of the use of the trading algorithm, the dispersion is lower, bounded around 10% of the value of the quota. As for the excess of average consumption, the consumption measured is 3.27% greater than the daily quota limit. This means that a community in which the excesses of consumption are penalized, like the one used in this work for the requirements of our contribution, benefits from the algorithm by reducing excess consumption by 2.37%.

## 8. Conclusions and Future Work

In this work, we propose a blockchain-based authorization system in order to perform an optimal control of the resources consumed in a smart community. This authorization system facilitates access to consumer information and resource trading.

We take advantage of the blockchain technology to build an information exchange platform used to secure transactions with cryptocurrencies, which in turn can be used to provide a flexible and secure platform to allow the provision of sensitive transactions, with privacy and reliability components.

On top of that we made two main contributions. First of all, we propose an authorization model by which devices belonging to the Wireless Sensor Networks can be authorized to view the consumption profile of a user. The proposed solution includes, as monitoring functions, secure data storage in the blockchain network and universal access to data by the Community gateway and as authorization functions, a procedure to allow the delegation of controller functions between Smart gateways.

In the second place, we propose a trustworthy resource trading through the provision of our Smart Community Token (SCT), as tradable asset unit representing the resource (energy, power, water) value in the blockchain. The defined SCT is compatible with PoA, a novel consensus strategy optimized for private networks with low computing power and oriented to a low consumption of resources.

We also introduce a new trading model, specific for exchanges of resources by cryptocurrencies, since it allows us to take advantage of the automatic generation of cryptocurrencies in the network and the theory of games in the auction stage.

We carried out the implementation of a prototype and its validation in various scenarios. The mechanism of proof of authority is measured, making a comparison to other state of the art solutions (PoW) in terms of power consumption and security concerns. We also carried out a performance evaluation for the resource monitoring proposal, considering the saturation level of the network according to the quantity and frequency of the information monitored and the computational cost of the proposed monitoring and delegation mechanisms, analysing the impact produced by the layer of security. Finally, we analyse the benefits of resource trading algorithm, based on Vickrey auction [43], in terms of reduction of dispersion. As conclusions of these analysis, the proposed system allows to monitor the information of resource consumption of a smart community environment for a discrete number of variables to monitor, consistent with most situations.

It is worth noting in the conclusions of this paper, that apart from the advantages clearly introduced by blockchain technology in terms of reliability of data, transparency in the distribution and exchange of resources, ease of establishment of transactions between untrusted peers and security in establishing authorization mechanisms, it also introduces a number of disadvantages. Firstly, high latency; it is observed that 20% of the transactions are reflected in the nodes after more than 20 s (generation time of two blocks). In addition, computational and storage overheads, resulting in a data generation rate of up to 3 GB per year, which must be stored in devices that could be of limited resources. Finally, there is the possibility, although remote, that a 51% attack will succeed and control the network, although the odds of this are at the level of other consensus-based security solutions. As future work, we will address the problem of sources of renewable energy generation within a smart community and how to adapt our model of resource monitoring and trading to contemplate prosumer users and the supply of surplus energy back to the grid. We would also like to advance in the generalization of the incentive management model to contemplate other aspects beyond energy saving, as a general collaborative model in a community of users. As smart contracts naturally lend themselves to machine-to-machine direct negotiations and transfer of goods, without human intervention, we will also improve the proposed mobile application to allow the user to establish their purchase preferences based on rules. These rules would be evaluated by the user’s smart home and would directly produce the automatic purchase or sale of resources if the given conditions are met.

## Figures and Tables

**Table 1:** Configuration parameters for private Ethereum network and PoA support.

Parameter	Value	Description
*eip150Block*	2	EIP150 since block #2: improvements for denial-of-service
*eip150Hash*	0x0	needed for fast sync
*eip155Block*	3	EIP155 since block #3: preventing replay attacks.
*eip158Block*	3	EIP158 since block #3: treating empty accounts as non-existent
*epoch_lng*	30000	Number of blocks after resetting pending votes
*block_period*	10 s	Minimum difference between two consecutive block’s timestamps
*extra-data*	65 bytes	Extra-data of each block enough to store the miner’s signature
*nonce*	{0xf, 0x0}	Vote on adding a new signer (0xf) or removing a signer (0x0)
*signer_count*	3	Number of authorized signers valid at a particular chain instance
*signer_limit*	2	Signer signs only one block out of *signer_limit* consecutive blocks for an in-turn signature
*difficulty*	{1, 2}	Block difficulty for blocks containing in-turn signatures (1) and out-of-turn signatures (2), with slight penalization

**Table 2:** Solution to attack methods in PoW and PoA.

Attack	PoW	PoA
Malicious contributor	Malicious miner needs 51% of hashing power for successful attach	Any signer may only mint 1 block out of every *K*. Damage is limited. Signer can be voted out.
Censoring contributor	Miner censoring blocks is penalized and their blocks are rarely included in the chain	Signers censoring blocks with negative votes are limited to 1 block out of *N/2* block.
Spamming contributor	Spamming transactions requires money to be spent on transaction fees	Signers injecting new vote proposals inside every block they mint is mitigated by placing a limit on the vote window.
Concurrent contributors	Concurrent block discovery is rare. In this case best fork is selected for chain continuation.	As at any point in time *N-K + 1* miners are allowed to mint. To avoid racing we add a small random “offset” to the time it releases a new block.

**Table 3:** Configuration parameters for performance evaluation.

Parameter	Value	Description
*num_SH*	40	Number of smart homes available in the smart community.
*block_period*	10 s	Minimum difference between two consecutive block’s timestamps
$M_{1}$	[0, N]	Length of essential data set for basic quota control.
$M_{2}$	[0, N]	Length of extended dataset for enhanced management.
*N*	10 KB	$\mathrm{Cardinality}\;\mathrm{of}\;\mathrm{the}\;\mathrm{complete}\;\mathrm{dataset}.\;\mathrm{We}\;\mathrm{consider}\;\mathrm{the}\;M_{1}\;\mathrm{and}\;M_{2}\;\mathrm{as}\;\mathrm{disjoint},\;\mathrm{being}\;N=M_{1}+M_{2}$.
*L*	variable	Number of records of essential data set samples which are simultaneously stored in the blockchain.
$T_{sample}$	*f(block_period)*	Sample generation period.
*G*	{0.25, 0.5, 1, 2}	Number of samples that will be generated by smart home in the time in which a block is generated.

**Table 4:** Computational cost in Gas for resource monitoring.

*L*	Solutions			
	Smonitoring	Monitoring	Sdelegation	Delegation
1	3.06 × 10^5^	8.44 × 10^4^	6.52 × 10^3^	1.12 × 10^3^
10^1^	3.45 × 10^6^	6.98 × 10^5^	6.18 × 10^4^	9.22 × 10^3^
10^2^	3.26 × 10^7^	6.02 × 10^6^	5.95 × 10^5^	9.17 × 10^4^
10^3^	3.11 × 10^8^	6.37 × 10^7^	5.72 × 10^6^	9.06 × 10^5^
10^4^	3.15 × 10^9^	6.25 × 10^8^	5.67 × 10^7^	8.87 × 10^6^
